# Epidemiology and antimicrobial resistance trends of *Acinetobacter* species in the United Arab Emirates: a retrospective analysis of 12 years of national AMR surveillance data

**DOI:** 10.3389/fpubh.2023.1245131

**Published:** 2024-01-04

**Authors:** Jens Thomsen, Najiba M. Abdulrazzaq, Hussain AlRand, The UAE AMR Surveillance Consortium, Abiola Senok, Dean B. Everett, Abiola Senok, Godfred A. Menezes, Carole Ayoub Moubareck

**Affiliations:** ^1^Abu Dhabi Public Health Center, Abu Dhabi, United Arab Emirates; ^2^Department of Pathology and Infectious Diseases, Khalifa University, Abu Dhabi, United Arab Emirates; ^3^Emirates Health Services Establishment, Dubai, United Arab Emirates; ^4^Public Health Sector, Ministry of Health and Prevention, Dubai, United Arab Emirates; ^5^Biotechnology Research Center, Khalifa University, Abu Dhabi, United Arab Emirates; ^6^Infection Research Unit, Khalifa University, Abu Dhabi, United Arab Emirates; ^7^College of Medicine, Mohammed Bin Rashid University of Medicine and Health Sciences, Dubai, United Arab Emirates; ^8^School of Dentistry, Cardiff University, Cardiff, United Kingdom; ^9^Department of Medical Microbiology and Immunology, RAK Medical and Health Sciences University, Ras Al Khaimah, United Arab Emirates; ^10^College of Natural and Health Sciences, Zayed University, Dubai, United Arab Emirates

**Keywords:** *Acinetobacter*, United Arab Emirates, multidrug-resistance, national surveillance, antimicrobial resistance

## Abstract

**Introduction:**

*Acinetobacter* spp., in particular *A. baumannii*, are opportunistic pathogens linked to nosocomial pneumonia (particularly ventilator-associated pneumonia), central-line catheter-associated blood stream infections, meningitis, urinary tract infections, surgical-site infections, and other types of wound infections. *A. baumannii* is able to acquire or upregulate various resistance determinants, making it frequently multidrug-resistant, and contributing to increased mortality and morbidity. Data on the epidemiology, levels, and trends of antimicrobial resistance of *Acinetobacter* spp. in clinical settings is scarce in the Gulf Cooperation Council (GCC) and Middle East and North Africa (MENA) regions.

**Methods:**

A retrospective 12-year analysis of 17,564 non-duplicate diagnostic *Acinetobacter* spp. isolates from the United Arab Emirates (UAE) was conducted. Data was generated at 317 surveillance sites by routine patient care during 2010–2021, collected by trained personnel and reported by participating surveillance sites to the UAE National AMR Surveillance program. Data analysis was conducted with WHONET.^1^

**Results:**

Species belonging to the *A. calcoaceticus-baumannii* complex were mostly reported (86.7%). They were most commonly isolated from urine (32.9%), sputum (29.0%), and soft tissue (25.1%). Resistance trends to antibiotics from different classes during the surveillance period showed a decreasing trend. Specifically, there was a significant decrease in resistance to imipenem, meropenem, and amikacin. Resistance was lowest among *Acinetobacter* species to both colistin and tigecycline. The percentages of multidrug-resistant (MDR) and possibly extensively drug-resistant (XDR) isolates was reduced by almost half between the beginning of the study in 2010 and its culmination in 2021. Carbapenem-resistant *Acinetobacter* spp. (CRAB) was associated with a higher mortality (RR: 5.7), a higher admission to ICU (RR 3.3), and an increased length of stay (LOS; 13 excess inpatient days per CRAB case), as compared to Carbapenem-susceptible *Acinetobacter* spp.

**Conclusion:**

Carbapenem-resistant *Acinetobacter* spp. are associated with poorer clinical outcomes, and higher associated costs, as compared to carbapenem-susceptible *Acinetobacter* spp. A decreasing trend of MDR *Acinetobacter* spp., as well as resistance to all antibiotic classes under surveillance was observed during 2010 to 2021. Further studies are needed to explore the reasons and underlying factors leading to this remarkable decrease of resistance over time.

## 1 Introduction

The extremely diversified *Acinetobacter* genus includes more than 50 species of nonpigmented, Gram-negative, oxidase-positive or oxidase-negative coccobacilli, the bulk of which are nonpathogenic, environmental organisms (1). The species *Acinetobacter baumannii*, *Acinetobacter calcoaceticus*, and *Acinetobacter lwoffii* are the most commonly detected, while *Acinetobacter haemolyticus*, *Acinetobacter johnsonii*, *Acinetobacter nosocomialis*, *Acinetobacter pittii*, and *Acinetobacter schindleri* are sporadically encountered (2–4). Typically, the four species *A. calcoaceticus*, *A. baumannii*, *A. pittii*, and *A. nosocomialis* form together the so called *A. calcoaceticus–A. baumannii* complex, being closely related and difficult to routinely distinguish; and recently, two new species, *Acinetobacter seifertii* and *Acinetobacter dijkshoorniae* were also included within that complex (5).

The 1960s and 1970s witnessed the emergence of infections caused by *Acinetobacter* species, coinciding with the use of sophisticated intensive care (6, 7). Initially thought of as a commensal opportunist with moderate virulence and little clinical significance, *Acinetobacter* infections increased in incidence and severity over the past few decades, an increase concomitant with rising prevalence of procedures including mechanical ventilation, central venous catheterization, and broad-spectrum antimicrobial therapy (8, 9). Nowadays, infections by *Acinetobacter* species, especially *A. baumannii*, are widely disseminated across hospitals, with highest density in intensive care units (ICUs), accounting for at least 20% of hospital-acquired infections in these wards (10), with estimates of resulting overall mortality exceeding 40% (11). In particular, *A. baumannii* group includes opportunistic pathogens that cause ventilator-associated pneumonia (VAP), central-line catheter-associated bloodstream infections (CLABSI), meningitis, urinary tract infections, surgical-site and wound infections, among others (12, 13). In one study, *A. baumannii* accounted for at least 9% of all Gram-negative infections and about 22% of infections in the ICU (14). Moreover, it is incriminated in over 7% of hospital-acquired pneumonia in the ICU and about 2% of nosocomial bloodstream infections (15). In a report issued in 2016 by the National Healthcare Safety Network in the US, the most frequent antimicrobial-resistant pathogens associated with health care-associated infections were reviewed. *Acinetobacter* species accounted for over 12% of VAP, 8.8% of CLABSI, 1.3% of catheter-associated urinary tract infections, and 1.3% of surgical site infections among gram-negative bacteria (16).

Along with the rise of *Acinetobacter* infections, major classes of antibiotics are threatened to lose their effectiveness against this pathogen, given its complex and varied resistance mechanisms (17). *Acinetobacter* exhibits exceptional capacity to retain a multidrug-resistant (MDR) phenotype, further complicating therapy, through a wide variety of pathways such as antibiotic-hydrolyzing enzymes, efflux pump alterations, impermeability, and antibiotic target mutations (18). As such, *Acinetobacter* spp. are capable of hydrolyzing β-lactams through the four different classes (A to D) of Ambler enzymes; produce aminoglycoside-modifying enzymes; expel multiple antibiotics by efflux pumps; alter carbapenem and aztreonam access through porin mutations; and modify key antibiotic targets like penicillin-binding proteins, DNA gyrase, and lipopolysaccharide (19–22). Accordingly, infections caused by *Acinetobacter* currently present a challenge to clinicians, and the available therapeutic options remain extremely limited (23). Due to accumulated mechanisms of resistance, *Acinetobacter* has been classified as MDR, extensively drug resistant (XDR) and pan-drug resistant (PDR), according to the published classification by Magiorakos et al. (24) for healthcare-associated, antimicrobial resistant bacteria. These phenotypes pose a real exertion to antimicrobial chemotherapy (25), and are associated with considerable mortality (26, 27).

In the Gulf Cooperation Council (GCC) and Middle East and North Africa (MENA) regions, accumulating data indicate the prevalence of MDR *Acinetobacter* infections. For example, Aedh and Colleagues (28) recently demonstrated alarming levels of resistance among MDR *Acinetobacter* in Saudi ICUs, with gentamicin and colistin being the most sensitive antibiotics. The rate of resistance to antibiotics from β-lactam, fluoroquinolone, and aminoglycoside groups was above 50%, while only trimethoprim-sulfamethoxazole was active against 50% of the isolates. Also in Qatar, different lineages of carbapenemase-producing, MDR *Acinetobacter* were reported (29). In Kuwait, independent research groups have previously documented expansion of MDR *Acinetobacter* across different hospitals, with polyclonal nature and transferrable resistance determinants (30–32). As far as the United Arab Emirates (UAE) is concerned, the first preliminary analysis of resistant *A. baumannii* from the Emirate of Dubai in 2021 reported multiple carbapenemase genes that have horizontally spread (33). An earlier study described MDR *Acinetobacter* with heterogeneous, sporadic types isolated from 5 different hospitals in Abu Dhabi (34). Moreover, in a local follow-up analysis from a single tertiary hospital at Al-Ain Emirate, a drop in imipenem susceptibility in *Acinetobacter* species from 99% in 2004 to only 32.5% in 2008 was noticed (35). Such evidence did shed a light on specific resistance mechanisms among local *Acinetobacter* species and created a background underscoring the need for further surveillance and control. However, large-scale, UAE-wide epidemiological studies of this group of bacteria are still lacking, and trends in antimicrobial resistance remain to be investigated. Of note, these trends have been increasing in reports from various regions (36–40), although some studies indicate a decreasing trend, such as that reported by Logan and colleagues (41), where cephalosporin-resistant *A. baumannii* decreased significantly between 2008 and 2012 in pediatric infections. The decrease was attributed to calls for improvement in infection control practices during that period, as well as to the concomitant release of an expert guidance on implementation of antimicrobial stewardship in critical care.

The UAE is a country in the GGC region well known for its cosmopolitan atmosphere, being a host for several nationalities and cultures, and a growing role as an international travel, tourism, finance, and health industry hub (42). Such blended and diversified population increases the risk for dissemination of resistant pathogens, and *Acinetobacter* are not an exception. Nevertheless, a consolidated, time-trend analysis of the evolution and changes in *Acinetobacter* resistance traits over a long period has not been previously realized in the UAE. While challenges in *Acinetobacter* species persist, given its nosocomial, resilient nature, longitudinal, retrospective, surveillance studies of such pathogen in a specific region remain necessary (17). Such studies highlight patterns and trends of infection and antibiotic resistance which eventually provide direction for strengthening infection control strategies in healthcare settings in this region (43), and should be beneficial to follow up *Acinetobacter* resistance patterns in the UAE.

The current investigation was realized to describe the longitudinal changes in *Acinetobacter* species resistance trends, as reported by the national antimicrobial resistance surveillance system that covers all the seven UAE Emirates. The specific objective of this follow-up study was to explore the nationwide status of *Acinetobacter* species resistance and evolving nosocomial patterns. It lays out the most prevalent *Acinetobacter* spp. observed at UAE healthcare facilities, along with the prevalence of their MDR, XDR, and PDR phenotypes, and represents the first documentation of a 12-year resistance portfolio in this pathogen across the whole country, from 2010 until 2021.

## 2 Materials and methods

### 2.1 Study design and data source

A multi-institutional retrospective observational study was conducted between 2010 and 2021 in the UAE using data extracted from the WHONET microbiology laboratory database software^2^ supported by the Global AMR Surveillance System protocol (GLASS, World Health Organization). Data was generated, collected, cleaned and analyzed through the UAE national AMR Surveillance program described by Thomsen et al. (44).

### 2.2 Identification and enrollment of national AMR surveillance sites

Starting 2010, UAE institutions were incorporated into the UAE national AMR surveillance program based on epidemiological needs assessment, readiness, and willingness of facilities to participate, availability of high-quality electronic AMR data, lab accreditation status, and qualification of staff. Hospitals, centers, and clinics representing all seven Emirates of the UAE joined the AMR surveillance network gradually over the years.

### 2.3 Bacterial population and variables of the study

All *Acinetobacter* spp. isolated from clinical samples during routine patient care by medical professionals in the National AMR surveillance sites, were included in this study from January 2010 to December 2021. Only the first isolate from each patient for each species per reporting period was included. Excluded from analysis were screening and quality control isolates, duplicate isolates, infection control related isolates, environmental isolates, and isolates from primary contaminated sources (pedibag).

The associated patient demographic information, clinical data, and microbiologic laboratory results were extracted from the national WHONET laboratory database software. The demographic variables included age, sex, nationality; clinical variables revealed the type of facility reporting the isolate (hospital/center/clinic), patient location, location type, specimen collection date, types of infection/specimen source; and microbiology variables revealed types of organism and antibiotic susceptibility testing results. The infection was considered to originate outside the center for outpatients or those presenting with the infection at the emergency department.

### 2.4 Bacterial identification

Bacterial identification was performed at the national AMR surveillance sites by medical professionals. The participating centers used at least one commercial, automated system for identification of bacteria, including VITEK^®^ (BioMérieux SA, Craponne, France), BD Phoenix^™^ (Becton Dickinson, New Jersey, United States) and MicroScan WalkAway (Beckman Coulter, Brea, CA, United States). Only one lab relied on manual systems like API^®^ (Analytical Profile Index. BioMérieux SA, Craponne, France) solely for identification.

### 2.5 Antimicrobial susceptibility testing

Antimicrobial susceptibility testing was performed at the National AMR surveillance sites using at least one commercial, automated system for routine antimicrobial susceptibility testing. Only two laboratories used manual testing methods (disk diffusion/Kirby Bauer). All labs followed CLSI guidelines for antimicrobial susceptibility testing of bacteria (CLSI-M100) (45). The criteria for interpretation of susceptibility testing results for tigecycline were adapted from the European Committee on Antimicrobial Susceptibility Testing (EUCAST 2022) guidelines (46). Any *Acinetobacter* spp. resistant to either imipenem, or meropenem, or both was considered as carbapenem-resistant *Acinetobacter* spp. (CRAB). To assess the multidrug-resistant (MDR) phenotype of the isolates the standard definition by Magiorakos et al. was used (24). To assess the extensively drug-resistant (XDR) and pandrug-resistant (PDR) phenotypes, a slightly modified version of the standard definition by Magiorakos et al. was used (24). Magiorakos’ et al. definitions for XDR and PDR phenotypes for *Acinetobacter* spp. includes 9 antimicrobial categories with 22 antibiotic agents. For technical reasons, associated costs, and local formulary requirements, participating laboratories would not routinely test all 22 antibiotics, i.e., some antibiotics were only very rarely (netilmicin, levofloxacin, ticarcillin/clavulanic acid, ampicillin/sulbactam, colistin, polymyxin B, tetracycline, doxycycline) or not at all (doripenem) tested. As such, the following, slightly modified definitions were used for ‘possible XDR’ and ‘possible PDR’ isolates (modifications highlighted in *italics*):

- ‘Possible XDR’: Non-susceptibility to at least one agent *routinely tested by clinical labs* in all but two or fewer antimicrobial categories, (i.e., bacterial isolates remain susceptible to only one or two categories).
- ‘Possible PDR’: Non-susceptibility to all agents *routinely tested by clinical labs* in all antimicrobial categories (i.e., no agents were tested as susceptible for that organism).

### 2.6 Statistical tests

Statistical significance of temporal trends for antimicrobial resistance percentages was calculated if data from at least 5 years was available. If fewer than 30 isolates per year were reported, or data was not available for all years within the considered period, trend analysis was not conducted. Statistical significance of trends is expressed as a *p*-value, calculated by a Chi-square for trend test (extended Mantel–Haenszel), using SPSS or Epi Info^™^. For testing the statistical significance of the difference for mortality and ICU admission a Chi^2^-test was used. For testing the statistical significance of the difference for length of stay (LOS), the weighted log-rank survival analysis was used. This was done to take care of differences in sample size between the groups. A *p*-value of less than 0.05 was considered statistically significant.

## 3 Results

### 3.1 Distribution of reporting sites for national AMR surveillance

**Figure 1:**
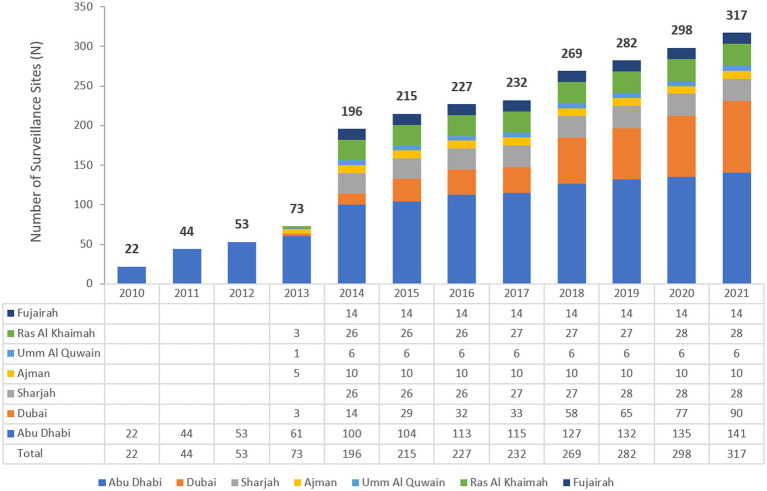
Number of AMR Surveillance sites by Emirate over the years of the surveillance period (2010–2021) in the UAE.

The UAE national AMR surveillance program was initiated in 2010 in the Abu Dhabi Emirate with 6 hospitals and 16 centers/clinics enrolled as AMR surveillance sites. Additional sites were recruited over the years, starting with 22 participating sites located only in the Emirate of Abu Dhabi in 2010, which is the first year during which the study was initiated, and reaching in 2021 a total of 317 surveillance sites, including 87 hospitals and 230 centers/clinics and representing all seven Emirates of the country. Figure 1 represents the distribution of reporting sites for National AMR Surveillance from 2010 to 2021, by year and Emirate.

### 3.2 Bacterial population

**Figure 2:**
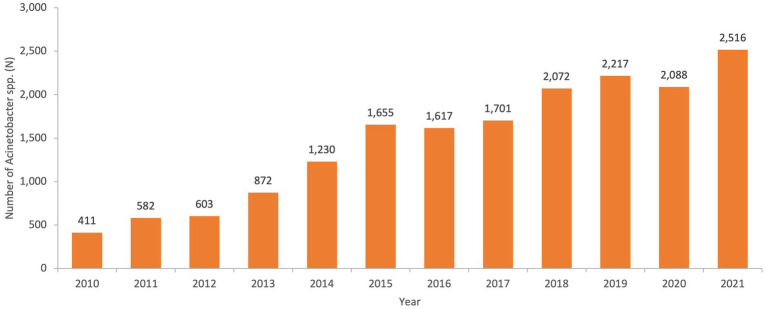
Numbers of non-repetitive *Acinetobacter* spp. isolated per year over the surveillance period in the UAE.

From 2010 to 2021, a total of 17,564 non-repetitive *Acinetobacter* spp. was isolated from an equivalent number of patients over the surveillance period. Figure 2 represents the number of *Acinetobacter* spp. isolates included per year.

### 3.3 Species distribution

Among the 17,564 *Acinetobacter* spp. analyzed, the vast majority belonged to the *A. calcoaceticus–A. baumannii* complex that includes *A. calcoaceticus*, *A. baumannii*, *A. pittii*, and *A. nosocomialis*. The overall percentages over the study period are shown in Table 1. More than 86.7% of the total number of *Acinetobacter* spp. collected during the surveillance period belongs to that complex.

**Table 1:** *Acinetobacter* species distribution as number and percentage of isolates across the study period (2010–2021).

Species distribution	Number of Isolates (N)	Percentage (%)
*Acinetobacter calcoaceticus-baumannii complex*	15,233	86.7
*Acinetobacter lwoffii*	800	4.6
*Acinetobacter junii*	376	2.2
*Acinetobacter haemolyticus*	198	1.1
*Acinetobacter johnsonii*	21	0.1
*Acinetobacter ursingii*	21	0.1
*Acinetobacter* spp.	915	5.2
**Total**	**17,564**	**100**

### 3.4 Distribution of *Acinetobacter* spp. patients by age, gender, nationality status, and Emirate

**Figure 3:**
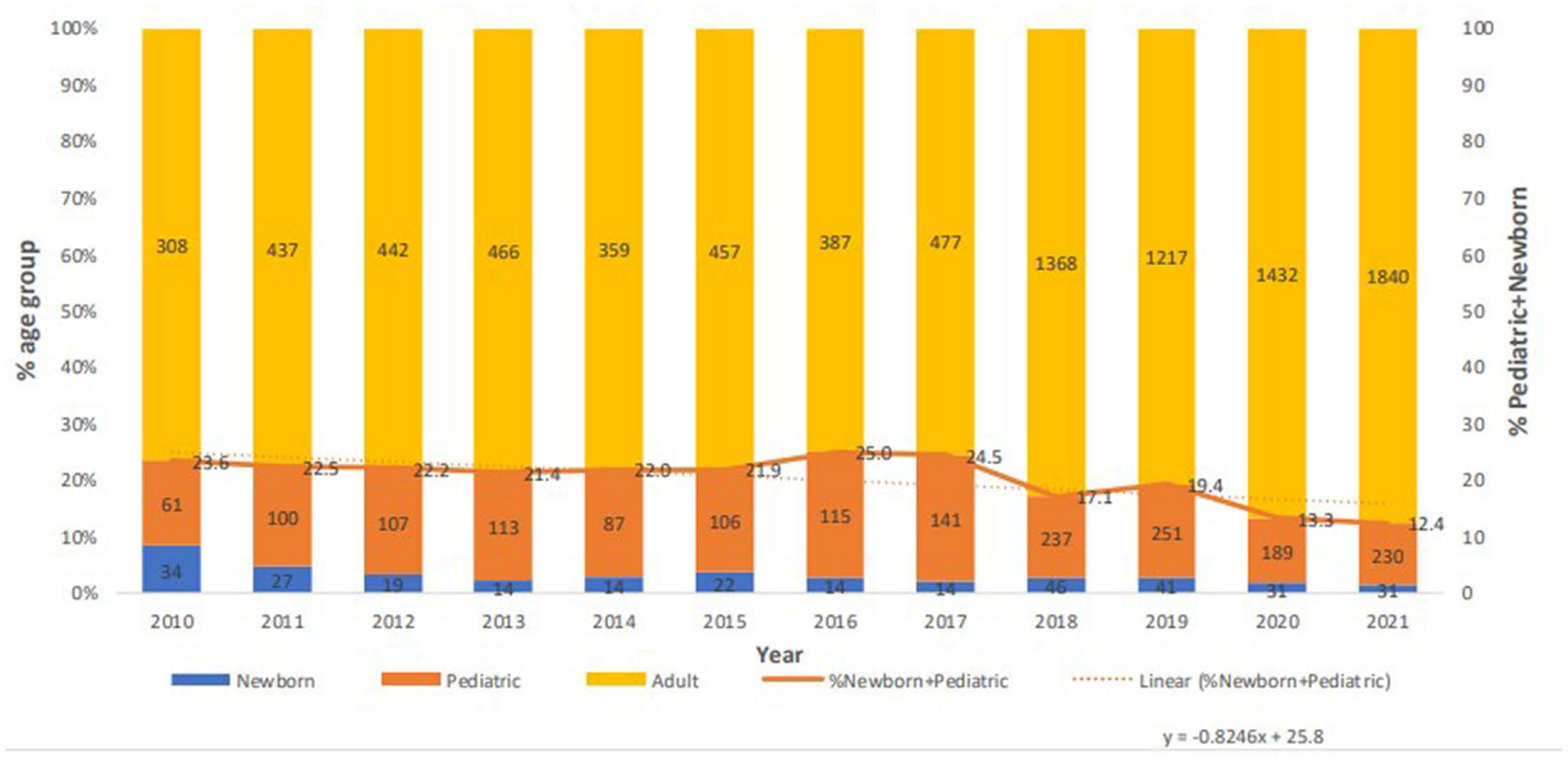
Age distribution of *Acinetobacter* spp. patients over the surveillance period per year.

*Acinetobacter* spp. strains were mostly associated with adults with a net decrease in the newborn and pediatric population since 2016 (Figure 3). Strains of *Acinetobacter* spp. were almost equally affecting males and females, with a 51% attributed to males.

**Figure 4:**
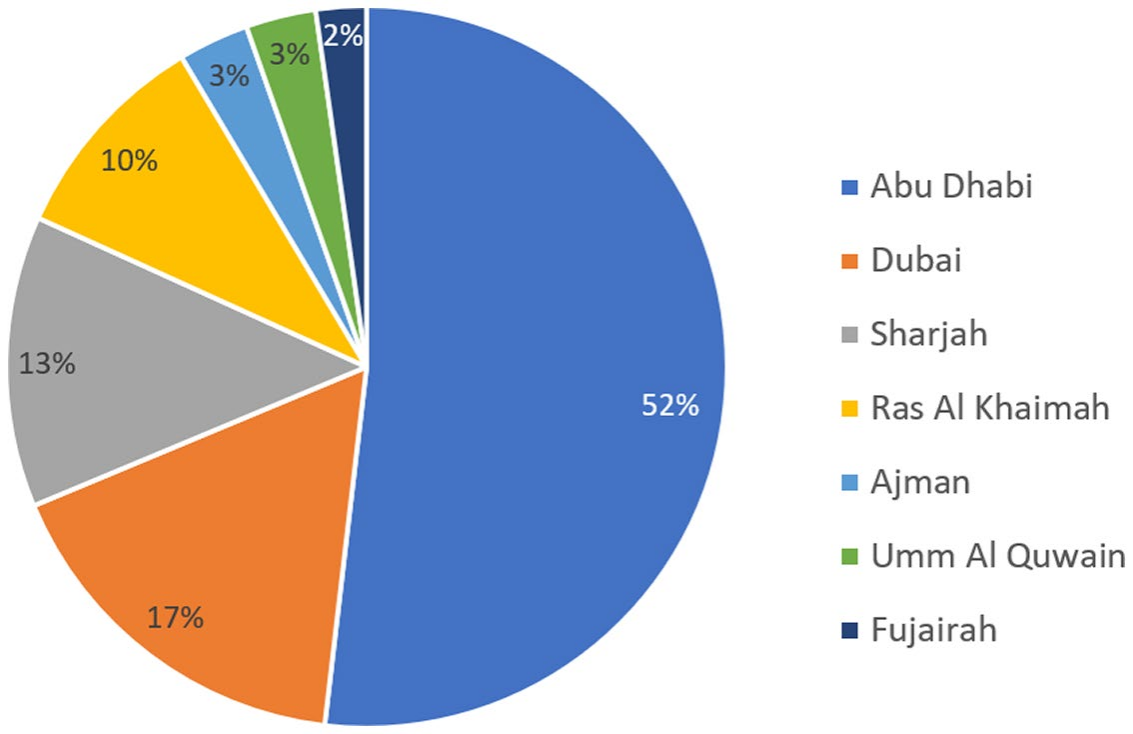
Distribution of patients carrying *Acinetobacter* spp. by Emirate over the surveillance period.

The nationality status of patients revealed a total of 23.1% of nationals and 36.3% of expatriates. For the remaining 40.6% of patients, the nationality status was missing. The majority of patients was detected in the Emirate of Abu Dhabi as shown in Figure 4, and those accounted for over half of the patients during which *Acinetobacter* isolates were recovered.

### 3.5 Distribution of *Acinetobacter* spp. by sample type group

**Figure 5:**
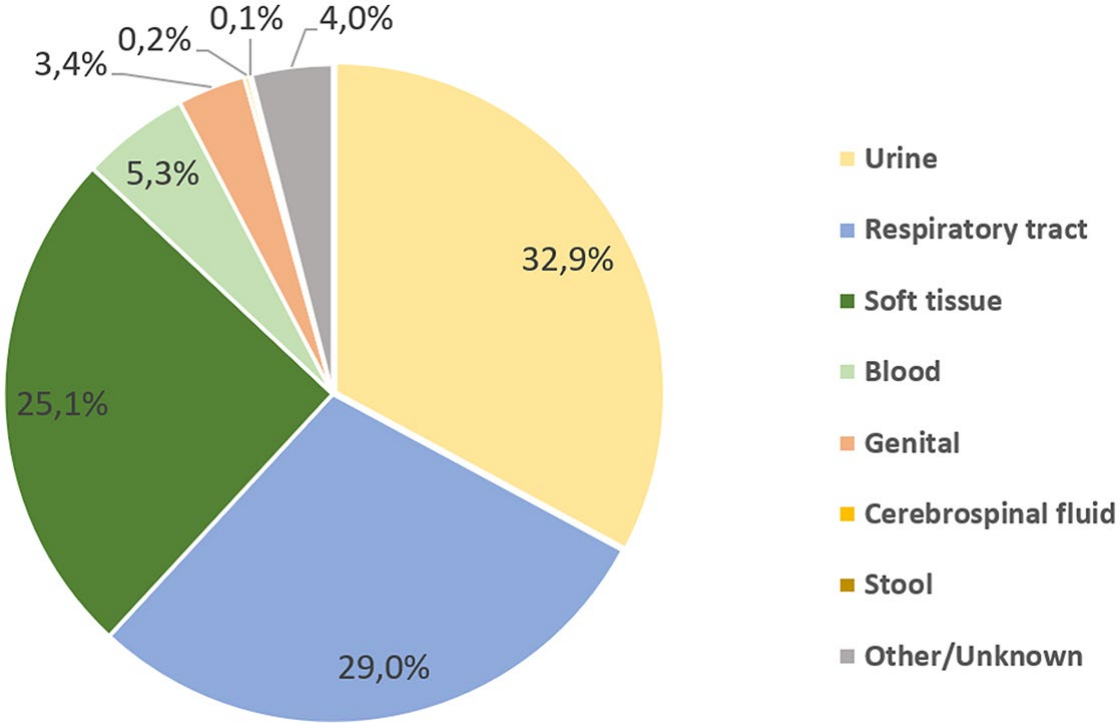
Distribution of *Acinetobacter* spp. by sample type group over the surveillance period.

Most of *Acinetobacter* spp. strains were isolated from urine (32.9%), followed by the respiratory tract samples (29.0%) and the soft tissue (25.1%) groups. The distribution of *Acinetobacter* isolates by clinical sample type is shown in Figure 5.

### 3.6 Distribution of *Acinetobacter* spp. by location (inpatients/outpatients) and department

Most strains of *Acinetobacter* spp. (65.2%) were detected in clinical settings (in hospitals rather than community settings and emergency wards) and were enrolled in general medical wards (26.8%) followed by ICUs (15.8%) and surgery departments (15.6%). A proportion of 34.8% of studied isolates originated in outpatient basis, being recovered either in the community, from outpatient centers and clinics, or in the hospital emergency departments.

### 3.7 Trend of antimicrobial susceptibility profiles of *Acinetobacter* spp.

**Figure 6:**
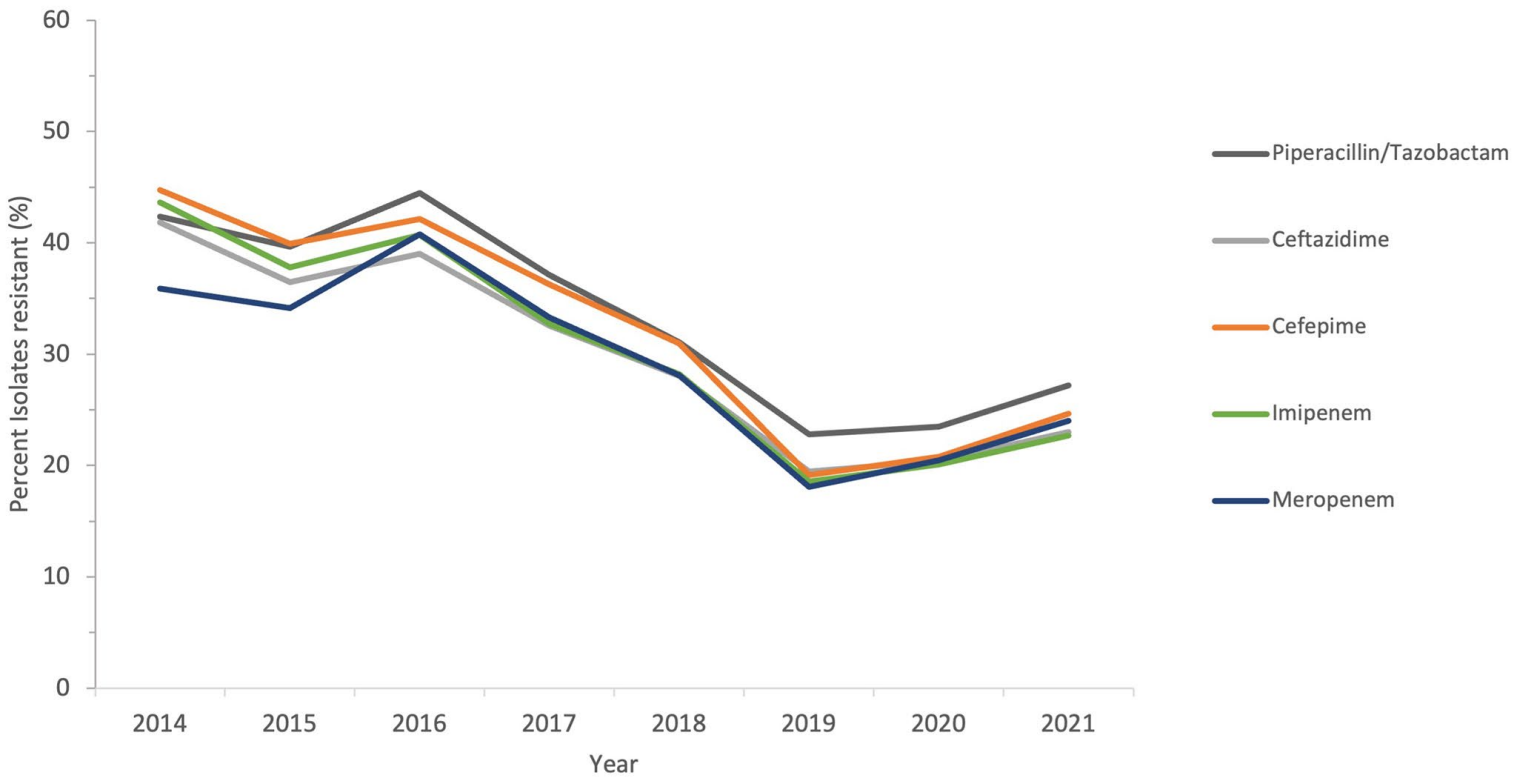
Susceptibility trends of *Acinetobacter* spp. to five different β-lactams over a selected period of the study (from 2014 to 2021).

**Figure 7:**
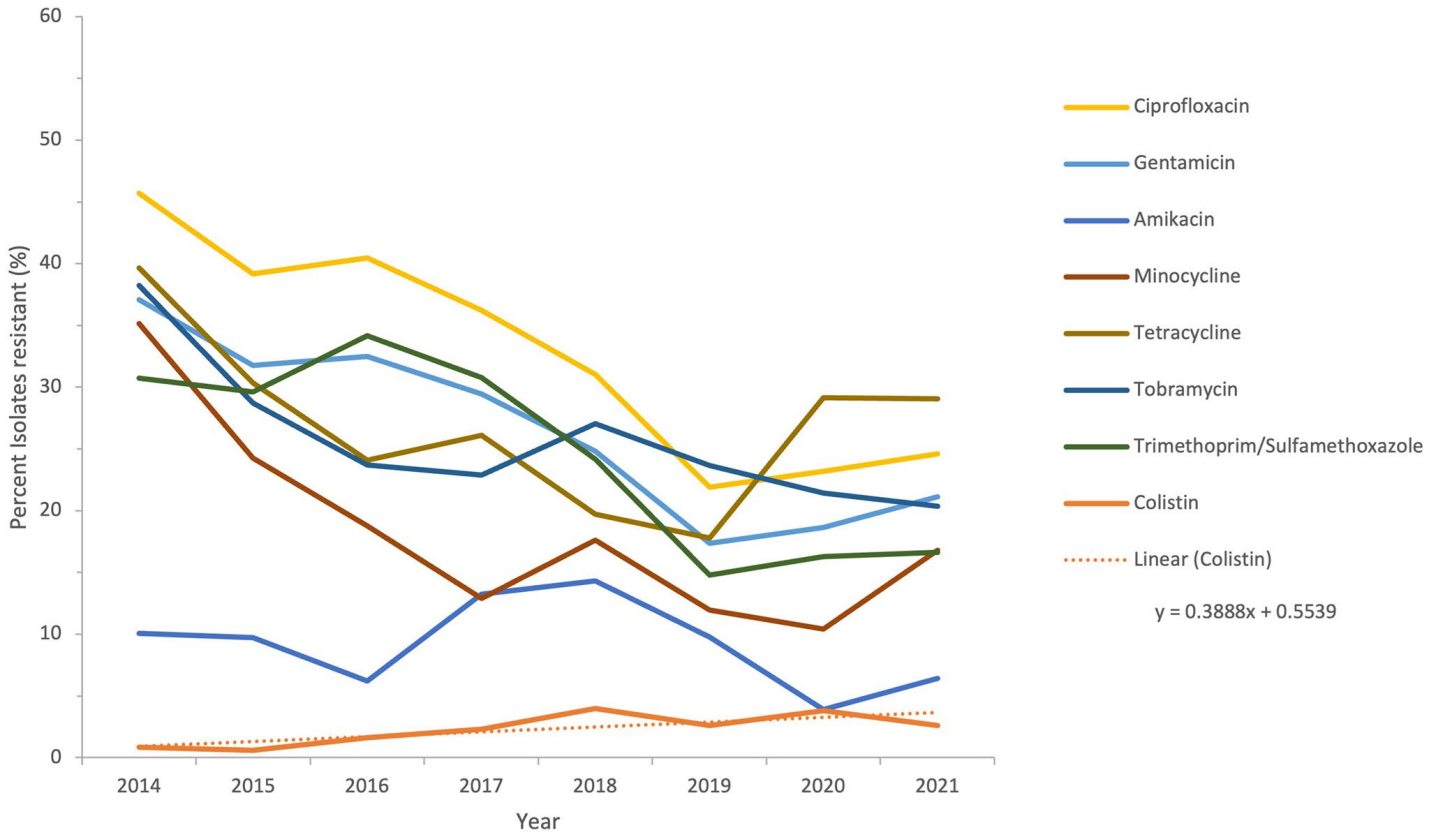
Susceptibility trends of *Acinetobacter* spp. to antibiotics other than β-lactams over a selected period of the study (from 2014 to 2021).

The sensitivity of all *Acinetobacter* spp. recovered during a period of the study, from 2014 to 2021, to antimicrobial agents from the β-lactam group and other groups is shown in Figures 6 and 7.

The resistance of isolates to multiple antibiotics showed a decreasing trend over the study period, as depicted from the general profiles in Figures 6 and 7. Specifically, resistance to imipenem and meropenem as well as to amikacin showed a statistically significant decrease over the past 12 years with a *p* value of zero. Resistance to colistin was low, showing an upper limit of 4% in 2018. Tigecycline resistance levels were the lowest, with maximum upper limit of 0.2% in 2019 and 2021, while they persisted at zero with isolates remaining highly sensitive to this antibiotic for all other study years.

**Figure 8:**
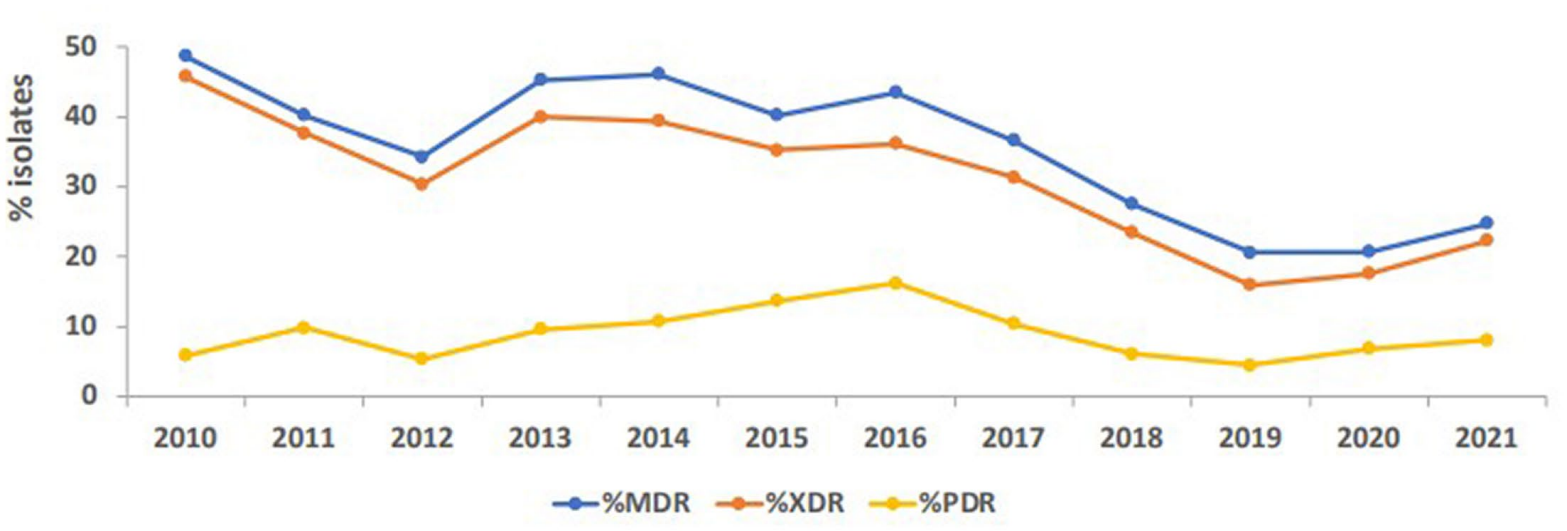
Trends of *Acinetobacter* spp. exhibiting multidrug-resistant (MDR), possible extensively drug-resistant (XDR) and possible pandrug-resistant (PDR) phenotypes over the study period.

The percentage of strains that exhibited MDR phenotype, as shown in Figure 8, being resistant to three or more classes of antibiotics, such as ciprofloxacin, gentamicin, tetracycline, trimethoprim/sulfamethoxazole, ceftazidime, piperacillin/tazobactam, imipenem, and meropenem, ranged between 48.7 in 2010 and 20.6 in 2019 and 2020, then raised again to 24.7 in 2021. The maximum percentage of possible XDR strains was reported in 2010 at 45.7%, and of possible PDR strains in 2016 at 16.2%. These figures were cut down to 22.3 and 8%, respectively, in 2021. As an overall trend, MDR, possible XDR, and possible PDR strains generally declined over the study period especially starting from the year 2016, as shown in Figure 8.

### 3.8 Mortality rate

A subgroup analysis including the nine clinical institutions that reported mortality was performed. In these institutions, a total of 4,306 patients were associated with non-CRAB of whom 272 patients died (mortality rate is 6.32%), while a total of 1,649 patients were associated with CRAB, of whom 593 patients died (mortality rate is 35.96%). The difference in mortality between CRAB patients (35.96%) and non-CRAB patients (6.32%) is statistically significant (RR 5.69, 95% C.I. 4.99, 6.50, *p* < 0.01).

### 3.9 Admission to intensive care unit

A total of 9,132 patients were associated with non-CRAB of whom 1,109 patients were admitted to ICU (ICU admission rate is 12.14%), while a total of 3,800 patients were associated with CRAB, of whom 1,510 patients were admitted to ICU (ICU admission rate: 39.740%). The difference in ICU admission rate is statistically significant (RR 3.27, 95% C.I. 3.06, 3.50, *p* < 0.01).

### 3.10 Length of stay (LOS)

A subgroup analysis including those patients for whom the date of admission as well as the date of discharge was known was performed. For patients in the non-CRAB group (*n* = 1,321) the median length of stay was 8 days, while for patients in the CRAB group (*n* = 715) the median length of stay was 21 days. Comparative differences in LOS were done using Kaplan–Meier curves and are shown graphically in Supplementary Figure 1. To assess if the observed difference in LOS was statistically significant, we performed a weighted log-rank test. This test showed that there was a significant difference in LOS between CRAB and non-CRAB patients (*p* < 0.001).

Based on a total of *n* = 4,921 patients associated with CRAB during the observation period (2010–2021), a total of 63,973 excess days of hospitalization is estimated attributable to CRAB. For the year 2021 only, a total of 7,384 excess hospitalization days is estimated attributable to CRAB.

## 4 Discussion

This is the first comprehensive analysis across the UAE that shows the significance and magnitude of *Acinetobacter* infections in clinical settings and the fluctuations in their antimicrobial resistance patterns. The present research utilized an extensive dataset collected over a considerable duration, allowing precise observation of subtle variations in antimicrobial resistance among *Acinetobacter*. This level of inclusive analysis has not been previously replicated in the country. The samples analyzed in this study consisted of non-repetitive *Acinetobacter* of laboratory confirmed identity and antibiotic resistance profile, indicating authenticity of the microbiological material used and accuracy of the generated data. Perhaps the most thought-provoking finding in this study is the observation of a decrease in antibiotic resistance in *Acinetobacter* over about 12 years, and this was evident despite an increase in the number of participating sites from 22 to 317, distributed across all the seven Emirates.

The UAE accommodates a diverse community comprising more than 200 nationalities. Emirati nationals make up approximately 10% of the overall population, highlighting the UAE’s status as one of the countries with a significant expatriate presence. Among the expatriate groups, Indians and Pakistanis represent the largest segments, accounting for 27.5 and 12.7% of the total population, respectively. However, our results show that about 23% of *Acinetobacter* samples were recovered from Emirati nationals, while 6.1 and 4.5% were recovered from Indian and Pakistani expatriates, respectively. These proportions of the total sample pool should be interpreted cautiously, since 40.6% of the samples attributed from patients for whom their nationality was not coded in the data, hence not available. With the expatriate-inclusive and multicultural setting, expected to prevail for the forthcoming years, the UAE may be an interesting niche to compare how trends of resistance in *Acinetobacter* differ by nationality, shedding a light on cultural and social factors contributing to resistance in a multidisciplinary research perspective, as previously suggested (47, 48). However, given that a massive 40.6% of our samples originated from patients with unknown nationality, this investigation could not be realized with our data, but remains tempting to explore. Moreover, the majority of patients (52%) from whom samples for the study were recovered were residents of the Emirate of Abu Dhabi, which also included the majority of participating centers (44.2%). Obviously, this conforms with the fact that Abu Dhabi has been the first Emirate to start AMR surveillance, and it also is the largest Emirate in terms of area, where is occupies over 80% of the nation’s land. However, Dubai, rather than Abu Dhabi, is the most populated Emirate, and samples from Dubai residents accounted for a much lower 17% only of those analyzed in this study. As such, these results must be cautiously interpreted.

As shown in Table 1, the majority of recovered species belong to *A. calcoaceticus-baumannii complex*, which alone occupied 86.7% of the total sample size. The remaining proportion was formed collectively from all other species, with *A. lwoffii* and *A. junii* accounting for 4.6 and 2.2% of all isolates collected throughout the study period, in addition to other species. Ubiquitous *Acinetobacter* species were initially thought of as commensals that are non-pathogenic to immunocompetent subjects (49); globally, reports on hospital outbreaks caused by *Acinetobacter* spp. have been mostly linked to *A. baumanni*, which is the most virulent species (50). However, several species of these ubiquitous bacteria have emerged as core pathogens in hospitalized patients and fomites, and can cause life-threatening nosocomial infections in compromised hosts (51). The spread of antimicrobial resistance to these species has further increased concerns regarding them and placed them as a special risk (52), especially in the ICU (53–55). With over 15% of different non-*baumannii* species isolated throughout the duration of the study, this may shed a light on the presence and long-term maintenance of these strains among patients and, accordingly, the need for precise documentation and tight control of different factors responsible for their dissemination in the UAE.

A consistent minimum of about 70% of species were recovered from adult patients, while pediatric samples declined from 2016 till the end of the study to reach about 12%. Reports of resistant *Acinetobacter* infections in children in different hospital settings have been previously published (56–58), even in children under 1 year with community acquisition of *A. baumanni* in their upper respiratory tract (59). This indicates possible epidemiological insights into the existence of *Acinetobacter* not only in critical settings, but also in non-serious community-based infections in the pediatric population. An in-depth analysis of *Acinetobacter* infections in infants and children in the UAE has not been yet realized. However, the decreasing rates of infection since 2016 are interesting, and warrant a further focused exploration of the epidemiology and resistance patterns of *Acinetobacter* in this population.

Urinary and respiratory samples contributed to the largest collection of studied samples, with percentages of 33 and 29%, respectively, from the bulk collection of *Acinetobacter*. Although described as a cause of catheter-associated urinary tract infections (60), most of the literature has focused on *Acinetobacter* in pneumonia and bloodstream infections; nevertheless, its role as a uropathogen cannot be neglected (61), and its isolation in urine samples in this study warrants attention. The second most common clinical specimen was from the respiratory tract, and this is in parallel to evidence indicating *Acinetobacter* abundance in respiratory samples with an incidence from 13 to 68% (62). Of note, the proportion of outpatient samples was about 35%, shedding a light on *Acinetobacter* reservoirs outside hospital settings, where they can be culprits in community-acquired pneumonia, infections in survivors from natural disasters, and infected war wounds (63). The actual presence of this organism in various environmental locations and being transferred to patients in the community is not possible to confer from our findings and needs further attention.

Over the full duration of the study, the rates of resistance of *Acinetobacter* to all tested antibiotics did not increase beyond 50%. This is in contrast to a recent report from the Gulf region, where in KSA, a neighboring country, the rates of resistance to all antibiotic in *A. baumannii* was above 50%, except for gentamycin and colistin (28). Also, in Oman, the rates of *A. baumannii* resistance to different antibiotics ranged from 50 to 83% (64). Previously, some studies detected the molecular epidemiology of *A. baumannii* resistance in the region, like detection of OXA-23 and OXA-24 from isolates collected from the Gulf Cooperation Council countries (65), detection of OXA-23, NDM-1 and GES-11 in isolates from Dubai (33), and detection of MDR and XDR strains in Abu Dhabi (34). In this study, a follow-up of resistance trends to different antibiotics consistently at a nationwide level showed that the rates of resistance declined from 2011 to 2021. This was true for all tested β-lactam antibiotics, whose resistance declined from a range of 40–50% at the beginning of the study to 20–30% in the last 3 years of follow-up. For other antibiotic classes, the trends of resistance were more heterogeneous in the first few years (due to smaller sample size), but all declined toward the end of the study. Perhaps the statistically significant decline in imipenem, meropenem, and amikacin is one of the most interesting findings of this analysis, and warrants to investigate the positive practices in the UAE that culminated into such a result. Similarly, the proportions of MDR and possible XDR strains were almost reduced by half, indicating that some isolates have regained their sensitivity or at least ceased being nonsusceptible over the course of the follow-up years. The mild increase in the levels of MDR, possible XDR, and possible PDR strains toward the end of the study period may have been affected by the COVID-19, as reported elsewhere (66, 67), and was probably driven by high rates of antimicrobial utilization and disruption of infection control measures occurring as collateral effects of the global pandemic (68). It is worth mentioning that trends in *Acinetobacter* species resistance were reported to have similar declining portraits in other parts of the world. For instance, a national study from the US showed a decline in carbapenem resistance (from 43 to 36%), MDR prevalence (from 49 to 36%), and XDR prevalence (from 21 to 10%) from 2010 to 2018. Similar epidemiological data of resistance decline in *Acinetobacter* exist from KSA (69), Germany (70), and Brazil (71). Hence, the decrease in resistance trends in *Acinetobacter* in the UAE mirrors other resistance trend observations in the region and elsewhere, and emphasize the need for continuing infection control and stewardship efforts and the development of new therapeutic options. While the trends for antimicrobial resistance to some antibiotics showed a slight rise after 2019, probably associated with factors related to the COVID-19 pandemic (72), the tends remained lower than those observed at the beginning of the study.

Looking into some specific antibiotics and the decrease in resistance among the studied isolates, it is noticeable that, for instance, imipenem and meropenem resistance rates in 2021 were less than 30%. This is much lower than rates of resistance reported in nearby countries like Jordan, where carbapenem resistance rate in 2022 was 99% (73). Moghnieh and Colleagues (74), in their narrative review on resistant Gram-negative pathogens in the region also described rates of carbapenem resistance in *Acinetobacter* above 80% not only in Jordan, but also in Lebanon and Iraq. The rates of carbapenem resistance in other countries including Turkey, Greece, Italy, and Spain, are much higher, with reported incidences of 50–80, 85, 60, and 45%, respectively (75, 76). For antibiotics from the non-β-lactam class, the highest resistance rates in 2021 were for tetracycline and ciprofloxacin, but both showed rates below 30%. This is in contrast to higher rates observed in Iran (tetracycline resistance 86%) (77), Egypt (ciprofloxacin resistance 42%) (78). However, the rates in this study were higher than those observed in Pakistan for ciprofloxacin (2.5%) and close to those for tetracycline (25%) (79). There are positive insights from the decline in resistance observed over the study duration, and comparison to other data from other countries reveals diverse resistance rates. However, the observed decline does not preclude the need for ongoing surveillance of *Acinetobacter* infections and continued assessment of effective prevention strategies, to build on the observed resistance mitigation for future attainments.

The two antibiotics tigecycline and colistin remained effective throughout the study period. According to previous evidence (80–84), combinations of these two antibiotics or combination of at least one of them with a third antibiotic have been used in treatment of MDR *Acinetobacter* infections, with variable success. However, both antibiotics remain among the most effective antimicrobial agents against *Acinetobacter* isolates *in vitro* (85), and their value needs to be preserved. As such, antimicrobial usage and consumption surveillance should aim at monitoring the use of colistin and tigecycyline, in presence of reports indicating resistance in *Acinetobacter* mainly mediated by the *tet*(X) gene against tigecycline (86, 87), and by the loss or modification of lipopolysaccharide or plasmid-encoded *mcr* genes against colistin (88, 89). The preservation of effectiveness of these two antibiotics in the UAE during this study, albeit with some rise in colistin resistance in 2018 and 2020, should provide an exemplar on maintaining the effect of last-resort antibiotics in clinical settings of high resistance.

Nevertheless, the mortality rate, according to our observations, was about 5.7-fold higher in patients infected with CRAB compared to those infected with non-CRAB *Acinetobacter* spp. Patients with an infection associated with CRAB were 3.3-fold more likely to be admitted to ICU, and their median length of stay was increased by 13 days, as compared to patients with non-CRAB infections. This is consistent with other findings that indicated high mortality rate and poor outcomes in patients with CRAB (90, 91) and highlights need for surveillance and control for better health outcomes.

## 5 Conclusion

This 12-year follow-up of the resistance trends in *Acinetobacter* species in the UAE indicated a decline in antimicrobial resistance and in proportions of *Acinetobacter* isolates with MDR and XDR profiles. The useful surveillance techniques, infection control strategies, and stewardship implemented over this span of time should be all reinforced. Further to these findings, continued epidemiological enquiry and genetic evolution analysis of *Acinetobacter* are required, to sustain the observed decline in resistance and to provide new strategies for prevention and control.

## Data availability statement

The datasets presented in this article are not readily available because the National AMR Surveillance database managed by the UAE Ministry of Health and Prevention (MOHAP) contains confidential health information. Requests to access the datasets should be directed to https://mohap.gov.ae/.

## Ethics statement

Ethical approval for this study was provided by the Ministry of Health and Prevention Research Ethics Committee (MOHAP/DXB-REC/ D.D.D/No.131 / 2021; MOHAP/DXB-REC/J.J.J./No. 86/2023), Dubai Scientific Research Ethics Committee (DSREC-GL17-2023), and Abu Dhabi Health Research and Technology Ethics Committee (DOH/ZHCD/2023/1316).

## Author contributions

CM, JT, NA, HA, DE, AS, and GM: conceptualization. JT, CM, NA, HA, DE, AS, GM, and The UAE AMR surveillance consortium: data collection. JT and CM: formal analysis. JT, CM, NA, HA, DE, AS, GM: data interpretation. CM and JT: manuscript preparation. CM, JT, NA, HA, DE, AS, and GM: manuscript review and editing. All authors contributed to the article and approved the submitted version.

## The UAE AMR surveillance consortium

**Table:** 

Nr	Name	Institution
1	Abiola Senok	College of Medicine, Mohammed Bin Rashid University of Medicine and Health Sciences, Dubai
2	Adnan Alatoom	Sheikh Shakhbout Medical City (SSMC), Abu Dhabi
3	Agnes-Sonnevend-Pal	University of Pécs, Pécs, Hungary
4	Ahmed Abdulkareem Al Hammadi	Tawam Hospital, Al Ain
5	Ahmed Elhag Ahmed	UAE University, College of Medicine and Health Sciences, Al Ain
6	Ahmed F. Yousef	Department of Biology, Center for Membranes and Advanced Water Technology, Khalifa University, Abu Dhabi
7	Alaa MM Enshasy	Dubai Health Authority, Dubai
8	Amal Mubarak Madhi	Abu Dhabi Public Health Center, Abu Dhabi
9	Amna AlBlooshi	Purelab, Al Ain
10	Andreas Podbielski	University Hospital Rostock, Rostock, Germany
11	Anju Nabi	Dubai Academic Health Corporation (DAHC), Dubai
12	Anup Shashikant Poddar	Al Sharq Hospital, Fujairah
13	Arun Kumar Jha	Danat Al Emarat Hospital, Abu Dhabi
14	Ayesha Abdulla Al Marzooqi	Abu Dhabi Public Health Center, Abu Dhabi
15	Bashir Aden	Khalifa University, Abu Dhabi
16	Carole Ayoub Moubareck	College of Natural and Health Sciences, Zayed University, Dubai
17	Dean Everett	Department of Pathology and Infectious Diseases, College of Medicine, Khalifa University, Abu Dhabi
18	Deeba Jafri	Purelab, Sheikh Khalifa Medical City, Ajman
19	Duckjin Hong	Sheikh Khalifa Specialty Hospital (SKSH) RAK
20	Emmanuel Fru Nsutebu	Sheikh Shakhbout Medical City, Abu Dhabi
21	Farah Ibrahim Al-Marzooq	United Arab Emirates University, Al Ain
22	Fatima Al Dhaheri	United Arab Emirates University, Al Ain
23	Fouzia Jabeen	Purelab, Sheikh Khalifa Hospital, Abu Dhabi
24	Francis Amirtharaj Selvaraj	Sheikh Khalifa Medical City (SKMC), Abu Dhabi
25	Ghada Abdel Wahab	Abu Dhabi Agriculture and Food Safety Authority, Abu Dhabi
26	Ghalia Abdul Khader Khoder	University of Sharjah, Sharjah
27	Gitanjali Avishkar Patil	NMC Specialty Hospital, Abu Dhabi
28	Godfred A. Menezes	Department of Medical Microbiology and Immunology, RAK Medical and Health Sciences University, Ras Al Khaimah
29	Hadayatullah Ghulam Muhammad	Emirates International Hospital, Al Ain
30	Hafiz Ahmad	RAK Hospital, Ras Al Khaimah
31	Hala Ahmed Fouad Ismail	PureLab, Al Qassimi Hospital, Sharjah
32	Hazim Khalifa	Department of Veterinary Medicine, UAE University, Al Ain
33	Husein Alzabi	Sheikh Khalifa General Hospital, Um al Quwain
34	Ibrahim Alsayed Mustafa Alhashami	Purelab, Al Qassimi Hospital, Sharjah
35	Imene Lazreg	University of Sharjah, Sharjah
36	Irfaan Akthar	Mediclinic City Hospital, Dubai
37	Jens Thomsen	Abu Dhabi Public Health Center, Abu Dhabi
38	John Stelling	WHONET, Boston, USA
39	Kaltham Ali Kayaf	Ministry of Climate Change & Environment (MOCCAE), Dubai
40	Kavita Diddi	Prime Hospital, Dubai
41	Krishnaprasad Ramabhadran	Burjeel Hospital, Abu Dhabi
42	Laila Al Dabal	Dubai Academic Health Corporation (DAHC, Dubai)
43	Laura Thomsen	University of Freiburg, Germany
44	Leili Chamani-Tabriz	Clemenceau Medical Center, Dubai
45	Madikay Senghore	Khalifa University, Abu Dhabi
46	Manal Abdel Fattah Ahmed	PureLab, Ras Al Khaimah
47	Maya Habous	Rashid Hospital, Dubai Academic Health Corporation, Dubai
48	Moeena Zain	American Hospital Dubai
49	Mohamud M. Sheek-Hussein	United Arab Emirates University, Al Ain
50	Monika Maheshwari	Al Zahra Hospital, Dubai
51	Monika Maheshwari	Medeor 24x7 Hospital, Dubai
52	Mubarak Saif Alfaresi	Zayed Military Hospital, Abu Dhabi
53	Mushtaq Khan	United Arab Emirates University, Al Ain
54	Najiba Abdulrazzaq	Al Kuwait Hospital, Emirates Health Services Establishment, Dubai
55	Nehad Nabeel Al Shirawi	Al Fujairah Hospital
56	Nesrin Helmy	Mediclinic Al Noor Hospital - Khalifa Street, Abu Dhabi
57	Pamela Fares Mrad	Abu Dhabi Public Health Center (ADPHC), Abu Dhabi
58	Pascal Frey	Berne University Hospital, Berne, Switzerland
59	Peter Nyasulu	Department of Global Health, Faculty of Medicine and Health Sciences, Stellenbosch University, South Africa
60	Prashant Nasa	NMC Specialty Hospital Al Nahda, Dubai
61	Rajeshwari T. A. Patil	Burjeel Medical City, Abu Dhabi
62	Rania El Lababidi	Dept. of Pharmacy Services, Cleveland Clinic Abu Dhabi
63	Ratna A. Kurahatti	NMC Royal Hospital Khalifa City A, Abu Dhabi
64	Riyaz Amirali Husain	Dubai Hospital, Dubai Academic Health Corporation, Dubai
65	Robert Lodu Serafino Wani Swaka	Sheikh Shakhbout Medical City, Abu Dhabi
66	Saeed Hussein	Erada Center for Treatment and Rehabilitation, Dubai
67	Sameh Soliman	University of Sharjah, Sharjah
68	Savitha Mudalagiriyappa	University Hospital Sharjah, Sharjah
69	Seema Oommen	Burjeel Medical City, Abu Dhabi
70	Shaikha Ghannam Alkaabi	Abu Dhabi Public Health Center, Abu Dhabi
71	Simantini Jog	Fakeeh University Hospital, Dubai
72	Simantini Jog	King’s College Hospital London Dubai Hills, Dubai
73	Siobhan O‘Sullivan	Khalifa University, Abu Dhabi
74	Somansu Basu	NMC Specialty Hospital, Al Ain
75	Stefan Weber	Purelab, Abu Dhabi
76	Sura Khamees Majeed	Al Gharbia Hospitals - Madinat Zayed Hospital
77	Syed Irfan Hussein Rizvi	Mediclinic City Hospital, Dubai
78	Tibor Pal	University of Pécs, Pécs, Hungary
79	Timothy Anthony Collyns	Tawam Hospital, Al Ain
80	Yassir Mohammed Eltahir Ali	Animal Wealth Sector, Abu Dhabi Agriculture and Food Safety Authority, Abu Dhabi
81	Yousuf Mustafa Naqvi	Department of Health Abu Dhabi (DoH), Abu Dhabi
82	Zahir Osman Babiker	Sheikh Shakhbout Medical City (SSMC), Abu Dhabi
83	Zulfa Omar Al Deesi	Latifa Maternity & Pediatric Hospital, Dubai

## Footnotes

^2^ https://whonet.org
