# Supplementary figures and images for: Epidemiology and antimicrobial resistance trends of Acinetobacter species in the United Arab Emirates: a retrospective analysis of 12 years of national AMR surveillance data
Source: Front Public Health. 2024 Jan 4;11:1245131. doi: 10.3389/fpubh.2023.1245131 (PMC10794577; doi:10.3389/fpubh.2023.1245131)

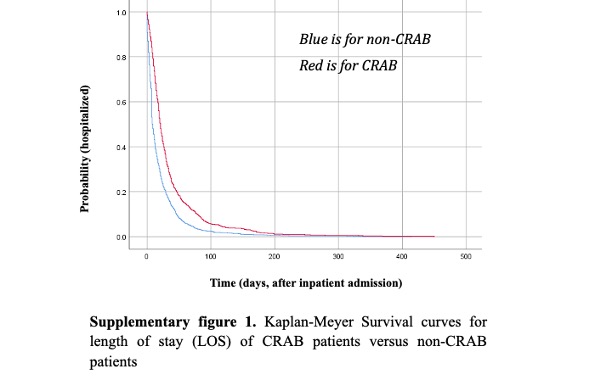

Supplement: Supplementary file 1 [file Image_1.jpg]
